# Improving structural variant clustering to reduce the negative effect of the breakpoint uncertainty problem

**DOI:** 10.1186/s12859-021-04374-3

**Published:** 2021-09-27

**Authors:** Jan Geryk, Alzbeta Zinkova, Iveta Zedníková, Halina Simková, Vlastimil Stenzl, Marie Korabecna

**Affiliations:** 1 Department of Biology and Medical Genetics, Second Faculty of Medicine, Charles University and University Hospital Motol, V Úvalu 84, 15006 Prague, Czech Republic; 2 Department of Biology and Medical Genetics, First Faculty of Medicine, Charles University and General University Hospital in Prague, Albertov 4, 128 00 Prague, Czech Republic; 3 Department of Forensic Genetics, Institute of Criminalistics, Strojnická 27, 170 89 Prague, Czech Republic

**Keywords:** Structural variants, Breakpoints uncertainty problem, Whole genome sequencing, Mendelian inheritance error, Constrained clustering

## Abstract

**Background:**

Structural variants (SVs) represent an important source of genetic variation. One of the most critical problems in their detection is breakpoint uncertainty associated with the inability to determine their exact genomic position. Breakpoint uncertainty is a characteristic issue of structural variants detected via short-read sequencing methods and complicates subsequent population analyses. The commonly used heuristic strategy reduces this issue by clustering/merging nearby structural variants of the same type before the data from individual samples are merged.

**Results:**

We compared the two most used dissimilarity measures for SV clustering in terms of Mendelian inheritance errors (MIE), kinship prediction, and deviation from Hardy–Weinberg equilibrium. We analyzed the occurrence of Mendelian-inconsistent SV clusters that can be collapsed into one Mendelian-consistent SV as a new measure of dataset consistency. We also developed a new method based on constrained clustering that explicitly identifies these types of clusters.

**Conclusions:**

We found that the dissimilarity measure based on the distance between SVs breakpoints produces slightly better results than the measure based on SVs overlap. This difference is evident in trivial and corrected clustering strategy, but not in constrained clustering strategy. However, constrained clustering strategy provided the best results in all aspects, regardless of the dissimilarity measure used.

**Supplementary Information:**

The online version contains supplementary material available at 10.1186/s12859-021-04374-3.

## Background

Structural variants (SVs) contribute significantly to the overall variation of the human genome. Their importance in diagnosing hereditary diseases has been recognized, but their detection remains a challenge in the modern bioinformatics field. In recent years, progress has been made in long-read sequencing, and the accuracy of SVs detection has improved [1–3]. However, the majority of available SV data was obtained by whole genome short-read sequencing (WGS) technologies [4, 5]. Thanks to their favorable price, these technologies are still widely used.

One of the important challenges within identification of SVs is the breakpoint uncertainty problem, which is often expressed in short-read sequencing technologies. The breakpoint uncertainty problem is defined as the inability to accurately determine the genomic position of SVs. Together with confidence bounds, the most probable position is often reported by SV detection programs. The accuracy of position determination is highly dependent on data quality—namely on read coverage and complexity of rearrangement around the genomic area where the SV occurs.

The SV population studies are most affected by the breakpoint uncertainty problem. Population analyses are typically based on many individual samples that must be merged to discover which SVs are common for which individuals. If the position of a single SV shared by two individuals is incorrectly detected in just one of the samples, this SV could then be incorrectly recorded as two different SVs in the resulting database. Consequently, this phenomenon can strongly affect studies of SV distribution within the genome, creating a false picture of many mutational hotspots.

To overcome this issue, heuristic clustering strategies try to estimate which groups of SVs actually correspond to a single SV with an incorrectly determined position within a group of individuals. Clusters of SVs of the same type occurring in close proximity are identified and then replaced by a single SV. To our knowledge, two main clustering strategies exist, differing in the dissimilarity measure used during the clustering procedure. The first strategy is based on the distance between the breakpoints of SVs and is implemented in the Linux tool *Survivor* [6]. The second strategy is based on the degree of overlap between SVs and has been used in several large population studies [4, 7].

To our knowledge, there is no study comparing the two above-mentioned clustering strategies or systematically examining the optimal clustering parameters represented by distance and degree of overlap. We will address both aspects within this study. We have also introduced a new measure of SV dataset quality based on the finding of a pattern of decomposed SVs within child-parent trios. Based on this measure, we proposed a new modification to the traditional methods that improves their performance.

## Results

In the presented article, we evaluated two dissimilarity measures ($D_{1}$, $D_{2}$) used in three clustering strategies (“trivial,” “corrected,” and “constrained”), as described in the “Methods.” In total, we examined six clustering scenarios.

**Fig. 1:**
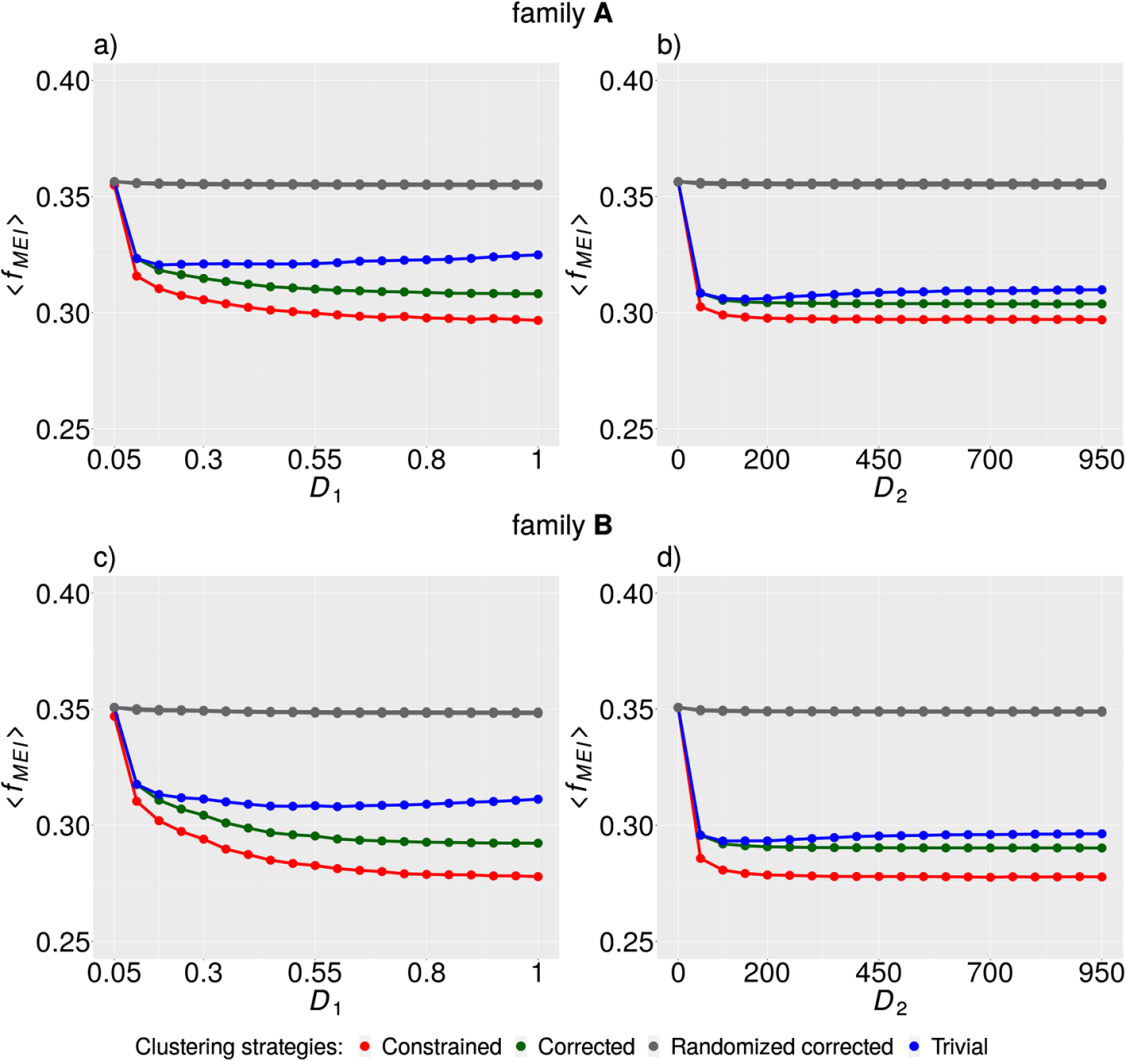
Average fraction of SVs corresponding to MEI $\left\langle {f_{MEI} } \right\rangle$. The dependence of $\left\langle {f_{MEI} } \right\rangle$ on the value of $D_{1}$ in family A (**a**) and family B (**c**) and the dependence of the same quantity on the value of $D_{2}$ in family A (**b**) and family B (**d**). All data points obtained by randomizations were concentrated in a very thin space, and so we represented the random ensemble by the (gray) curve corresponding to minimum values of $\left\langle {f_{MEI} } \right\rangle$ obtained for every value of $D_{1\left( 2 \right)}$

The first measure we used for dataset quality evaluation was the average fraction of SVs with Mendelian error $\left\langle {f_{MEI} } \right\rangle$, which measures the average fraction of erroneous genotype configurations within parent–child trios for both examined families (Fig. 1). We can see the general pattern valid for both families and all investigated clustering strategies except for the random model ensemble. The value of $\left\langle {f_{MEI} } \right\rangle$ begins to decline rapidly, then the rate of decline slows to a constant value, or in the case of trivial strategy, starts to grow slowly. We can see that value of $\left\langle {f_{MEI} } \right\rangle$ is roughly constant for $D_{1} \ge 0.8$ or $D_{2} \ge 150$ in the case of the corrected and constrained clustering strategies and both families (Fig. 1a, c). The constrained clustering strategy exhibits the best performance in comparison with other strategies.

**Fig. 2:**
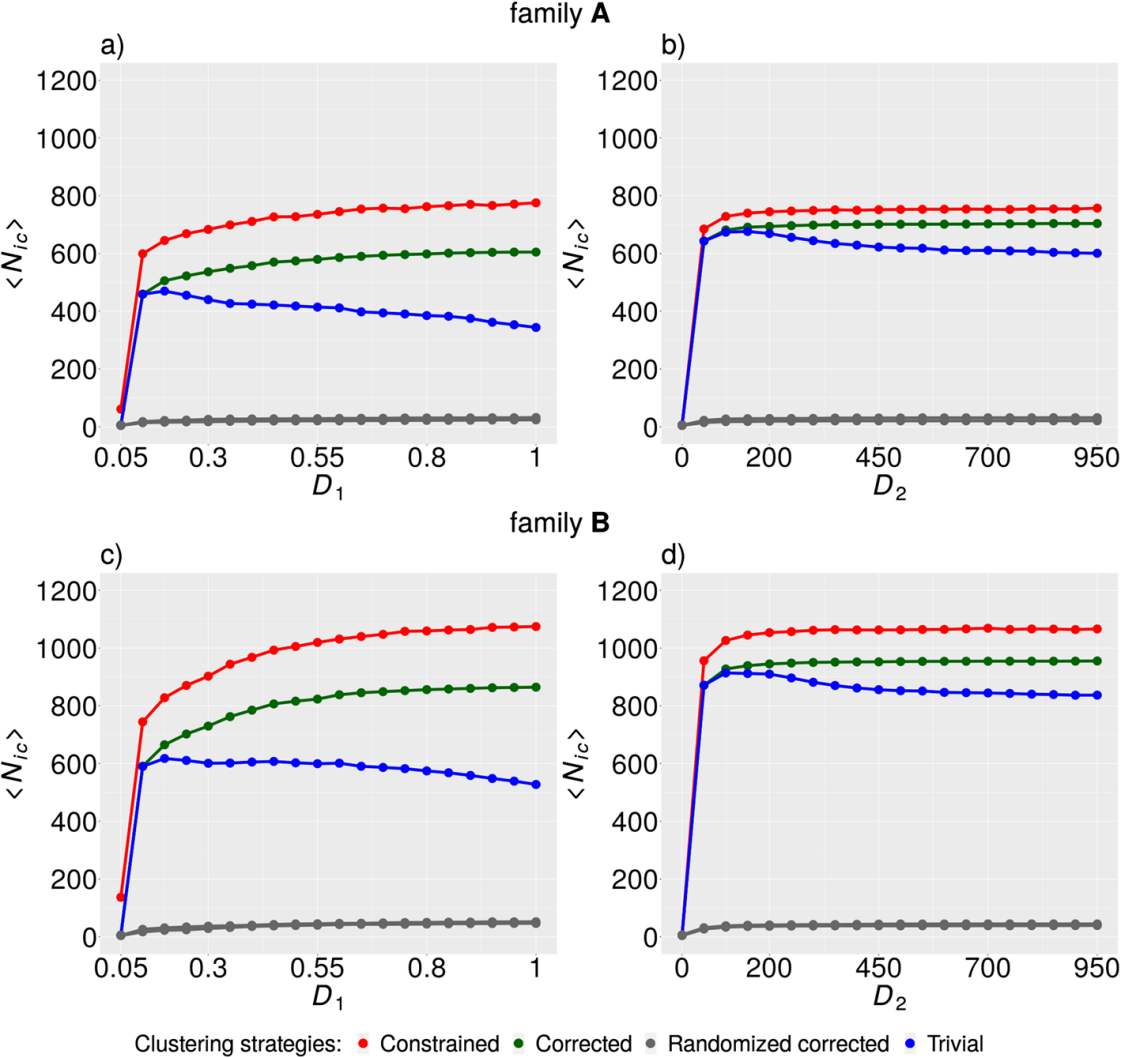
Average number of Mendelian-consistent SVs resulting from merging SV clusters exhibiting Mendelian errors $\left\langle {N_{ic} } \right\rangle$. The dependence of $\left\langle {N_{ic} } \right\rangle$ on the value of $D_{1}$ in family A (**a**) and family B (**c**) and the dependence of the same quantity on the value of $D_{2}$ in family A (**b**) and family B (**d**). Random ensemble is represented by the (gray) curve corresponding to maximum values of $\left\langle {N_{ic} } \right\rangle$ obtained for every value of $D_{1\left( 2 \right)}$

For the second measure, we used the average number of Mendelian-consistent SVs resulting from the merging of SV clusters exhibiting Mendelian errors $\left\langle {N_{ic} } \right\rangle$. We observed approximately inverse behavior of $\left\langle {N_{ic} } \right\rangle$ with respect to the $\left\langle {f_{MEI} } \right\rangle$ (Fig. 2). The largest increase of $\left\langle {N_{ic} } \right\rangle$ had already been observed at the lowest values of $D_{1}$ and $D_{2}$. This observation implies that most Mendelian-erroneous clusters were collapsed into a single Mendelian-consistent SV at the lowest investigated values of both dissimilarity measures,$D_{1} = 0.05$ and $D_{2} = 50$. The random model exhibited only very small values of $\left\langle {N_{ic} } \right\rangle$ in comparison with real data. This fact clearly shows that the pattern of decomposed SVs represents a statistically significant feature of the data. As in the previous case, $\left\langle {N_{ic} } \right\rangle$ was roughly constant for value $D_{1} \ge 0.8$ or $D_{2} \ge 150$ in the case of the corrected and the constrained clustering strategies and both families. The constrained clustering strategy exhibited the best performance in comparison with other strategies.

The decline of $\left\langle {N_{ic} } \right\rangle$ in the case of the trivial strategy (Fig. 2a, c) is explained by the fact that clusters grow with increasing value of $D_{1\left( 2 \right)}$, and thus the probability of a merging-incompatible SV pair occurrence within an identical cluster also increases. After a merging-incompatible pair of SVs occurs in a cluster for some value of $D_{1\left( 2 \right)} = x$, the SVs from that cluster will never be merged again for values of $D_{1\left( 2 \right)} > x$. By this mechanism, the SVs that collapsed at smaller values of $D_{1\left( 2 \right)}$ can be decomposed back at higher values of $D_{1\left( 2 \right)}$ and thus contribute to the decline of $\left\langle {N_{ic} } \right\rangle$.

We also computed the average number of Mendelian inheritance errors (MIEs) resulting from merging SV clusters without any MEIs. We found only negligible, constant amounts $\left( { < 10} \right)$ of this type of SV in all clustering scenarios, so we did not present this result in a graphical form.

**Fig. 3:**
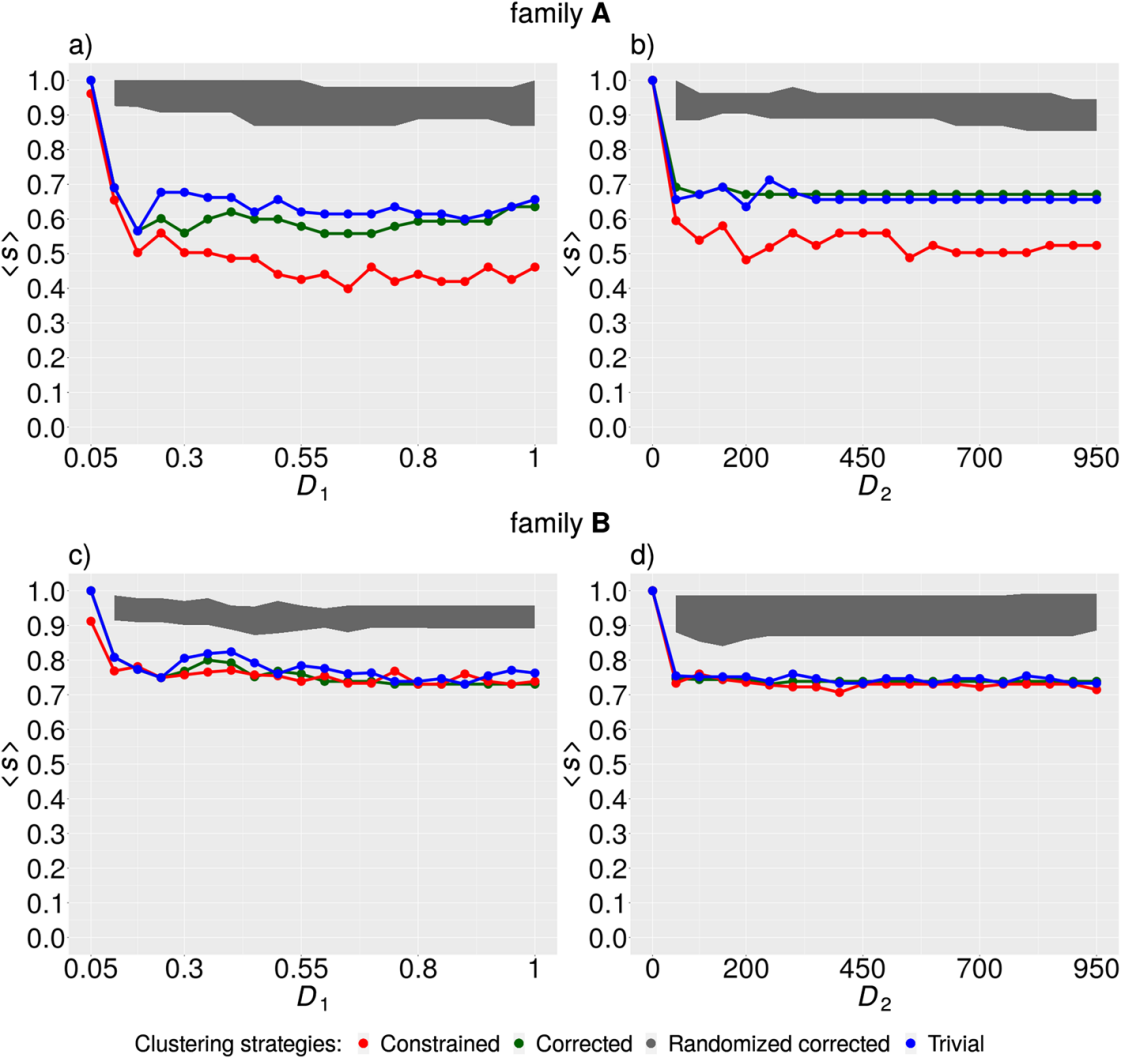
Average degree of separation between kinship categories achieved by the Loiselle kinship estimator $\left\langle s \right\rangle$. The dependence of $\left\langle s \right\rangle$ on the value of $D_{1}$ in family A (**a**) and family B (**c**) and the dependence of the same quantity on the value of $D_{2}$ in family A (**b**) and family B (**d**). Random ensemble is represented by the gray area defined from above by maximum values of $s$ and from below by minimum values of $\left\langle s \right\rangle$

The average degree of separation between different kinship categories achieved by the kinship estimator $\left\langle s \right\rangle$ was used as the third measure. In this case, the average was taken over all kinship categories, in contrast to the previous two approaches where the average was taken over all family trios as described in the “Methods.” For purposes of kinship prediction, we used only Mendelian-consistent SVs that resulted from collapsing SV clusters exhibiting Mendelian error. Our goal here was to rule out whether these SVs were incorrectly assembled by the new constrained clustering method and thus predicted the kinship less well than other methods. The constrained clustering method exhibited the best performance (smallest values of $\left\langle s \right\rangle$) in family ${\mathbf{A}}$ (Fig. 3). In family ${\mathbf{B}}$, constrained clustering performed similarly to the other methods (Fig. 3).

**Fig. 4:**
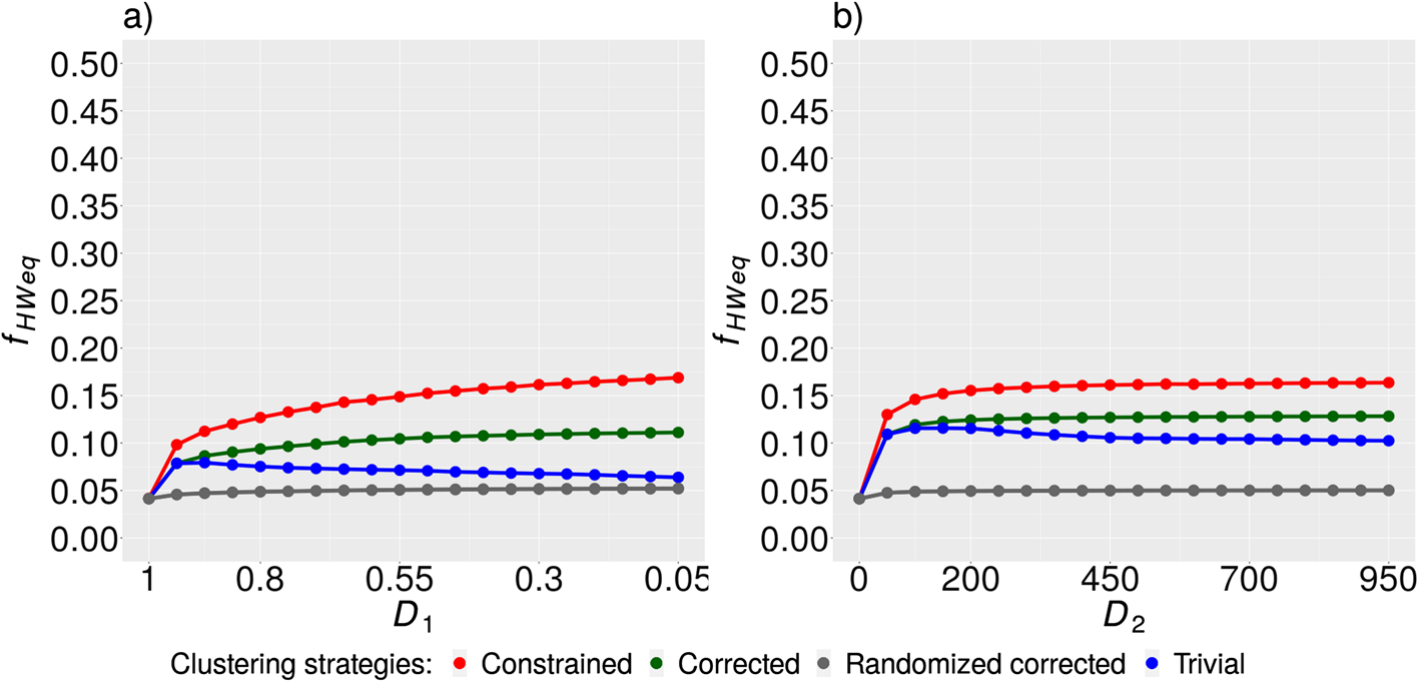
Fraction of SVs in Hardy–Weinberg equilibrium $f_{HWeq}$. The dependence of $f_{HWeq}$ on the value of $D_{1}$ (**a**) and the dependence of the same quantity on the value of $D_{2}$ (**b**). Random ensemble is represented by the (gray) curve corresponding to maximum values of $f_{HWeq}$ obtained for every value of $D_{1\left( 2 \right)}$

The last measure we used for our comparison of clustering scenarios was the fraction of SVs in Hardy–Weinberg equilibrium $f_{HWeq}$, which was computed using the data subset of unrelated individuals. The constrained clustering method gave the best results in this case (Fig. 4). The function $f_{HWeq}$ corresponding to this method was roughly constant for $D_{2} \ge 200$, but in the case of $D_{1}$, it did not show a tendency to stabilize in contrast to the corrected strategy (Fig. 4a).

**Fig. 5:**
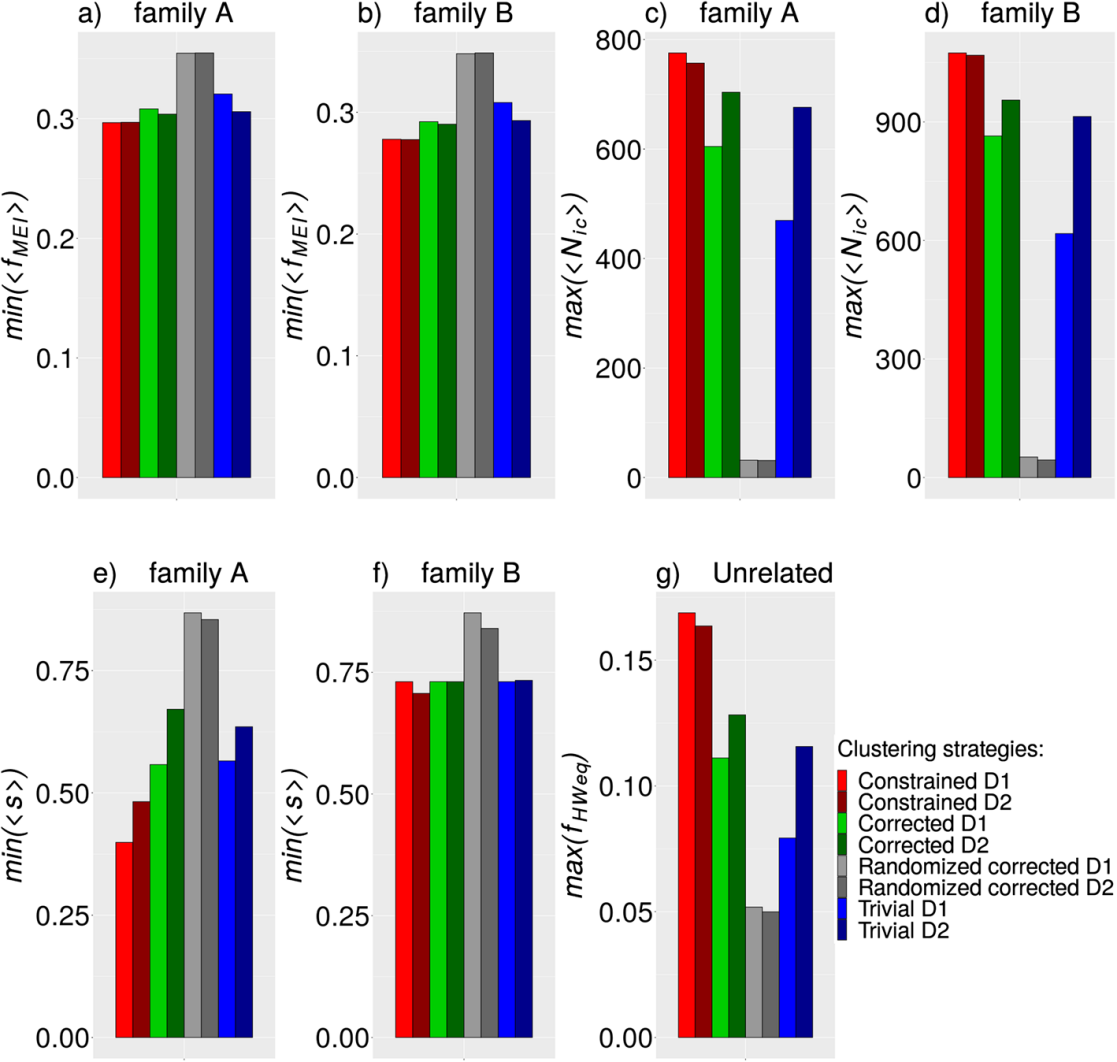
Maximum/minimum values of studied quantities corresponding to best performance. The minimum achieved values of $\left\langle {f_{MEI} } \right\rangle$ for all clustering strategies with combination of $D_{1}$ or $D_{2}$ values are visualized on sub-figures (**a**) and (**b**), the maximum achieved values of $\left\langle {N_{ic} } \right\rangle$ are visualized on sub-figures (**c**) and (**d**), the minimum achieved values of $\left\langle s \right\rangle$ are visualized on sub-figures (**e**) and (**f**) and the maximum achieved values of $f_{HWeq}$ are visualized on sub-figure (**g**)

To compare the performance of dissimilarity measures $D_{1}$ and $D_{2}$, we plotted the maximum/minimum achieved values of the investigated quantities when using $D_{1}$ or $D_{2}$ (Fig. 5). The difference between $D_{1}$ and $D_{2}$ is very small in the constrained clustering strategy if we focus on the three most important variables for benchmarking purposes (Fig. 5a–d, g). Conversely, in the corrected and trivial strategies, we see a more significant difference between the $D_{1}$ and $D_{2}$ measures, especially when we focus on the quantities $\left\langle {N_{ic} } \right\rangle$ and $f_{HWeq}$ (Fig. 5c, d, g). It is also clear from Fig. 5 that $D_{2}$ produces consistently better results than $D_{1}$ in both the corrected and trivial strategies (Fig. 5a–d, g). In the case of the variables $\left\langle {N_{ic} } \right\rangle$ and $\left\langle {f_{MEI} } \right\rangle$, it is possible to statistically test the difference between $D_{1}$ and $D_{2}$ because we have a group of $N_{ic}$ and $f_{MEI}$ values associated with individual trios. We observed statistical significance only for $N_{ic}$ in the trivial strategy and both families (*p* < 0.0147, Wilcox test) and in the corrected strategy and family A (*p* = 0.0143, Wilcox test). All other comparisons between $D_{1}$ and $D_{2}$ within individual strategies were not statistically significant.

## Discussion

The main difference between $D_{1}$ and $D_{2}$ is that $D_{2}$ may be relatively low for a pair of disjointed SVs. Conversely, $D_{1} = 1$ for any disjointed SV pair, a maximum value obviously never used as a threshold for clustering purposes. Consequently, the disjointed SV pairs will never be clustered in $D_{1} ,$ despite being in close proximity. The higher maximum value of $\left\langle {N_{ic} } \right\rangle$ in $D_{2}$ and the simple clustering strategies (Fig. 5c, d) can be explained by the existence of disjointed SV pairs resulting in a Mendelian-consistent single SV after merging. These pairs are clustered when using $D_{2}$ but not when using $D_{1}$.

In constrained clustering strategy, the maximum value of $\left\langle {N_{ic} } \right\rangle$ is slightly higher for $D_{1}$ than $D_{2}$ in contrast to the simple clustering strategies (Fig. 5c, d). This is related to the combinatorial search of SV groups that can be merged into single Mendelian-consistent SV in the initial phase of the algorithm. With higher $D_{1}$ values, the space for a combinatorial search can increase more quickly for $D_{1}$ than for $D_{2} ,$ assuming the existence of very large SVs. The greater the combinatorial search space, the greater the probability the algorithm will find Mendelian-consistent configurations by chance. Based on the fact that very large SVs are more likely artifacts, we can say that $D_{2}$ dissimilarity measure is more robust with respect to this undesirable phenomenon.

According to the MEI-based quantities that we considered the most important, it can be deduced from our data that the best performance is obtained for values $D_{1} \ge 0.8$ or $D_{2} \ge 150$ for both corrected and constrained clustering strategies. We must emphasize here that these thresholds may be dependent on the size of the data and the technologies used to obtain them. However, the measures presented in this article can be used to find optimal values of arbitrarily defined dissimilarity measures in any future study addressing the problem of merging individual SV profiles, assuming the presence of parent–child trios.

We also showed that our new constrained clustering strategy produced the best results in terms of all measures examined. It can be argued that MEI-based quantities, especially $N_{ic}$, have limited informative values in this case because maximization of $N_{ic}$ is implicitly included in the constrained clustering algorithm. Therefore, it is important to use other independent quantities for the purposes of algorithm assessment. We used kinship prediction and the fraction of SVs in Hardy–Weinberg equilibrium for these purposes and showed that the constrained clustering strategy exhibits superior performance in this case as well. However, other potential quantities should be used for a more accurate evaluation of this new method.

We observed a statistically significant enrichment of the pattern of decomposed SVs during the evaluation of conventional clustering strategies (Fig. 2). Based on this, the implementation of explicit identification of decomposed SVs can be seen as a test of how extensive the pattern appears in the data. We hypothesize that other patterns potentially exist in the SV data whose relevance for SV reconstruction can be tested using a random model ensemble. The method of explicit identification of decomposed SVs is mainly beneficial for related samples. However, the incorporation of cannot-link constraints representing merging-incompatible SVs within our constrained clustering strategy seems to also be beneficial for unrelated samples (Fig. 4). Future research is needed to elucidate the role of these two main components of the constrained clustering approach.

## Conclusions

This paper explored different strategies for SV clustering designed to reduce the impact of the breakpoint uncertainty problem when merging different WGS samples. Two dissimilarity measures along with three clustering strategies were benchmarked. We found that the $D_{2}$ dissimilarity measure performed slightly better than $D_{1}$ when combined with simple clustering strategies. We also presented the new constrained clustering strategy based on the identification of decomposed SV clusters within parent–child trios and demonstrated the best performance of this method.

## Methods

### Data preparation

Whole genome sequencing of all samples was performed on the NovaSeq 6000 platform with a target coverage of 30 by the commercial provider. The NEBNext Ultra DNA Library Prep Kit was used for library preparation.

### Dataset structure

We analyzed in total 124 WGS samples, all corresponding to healthy Czech individuals. Out of them, 10 samples formed a three-generational family with relationship coefficients of $\left( {0, 0.125, 0.25, 0.5} \right)$. In the article, we refer to this family as family ${\mathbf{A}}$. Another 12 samples within our dataset formed a different three-generational family with relationship coefficients of ($0, 0.0625, 0.125, 0.25, 0.5$). In the article, we refer to this family as family ${\mathbf{B}}$. The remaining 102 samples were unrelated individuals.

### Data pre-processing and SV identification

The fastq files corresponding to individual probands were processed by the generic data pre-processing pipeline published by Broad Institute [8]. The pipeline aligns sequences within fastq files to the hg38 genome build, performs base recalibration, and produces analysis-ready bam files. We also performed quality control using another pipeline published by Broad Institute [9]. Based on quality analysis, we excluded three samples with very low coverage.

The SVs were detected using Manta [10]. The vcf files containing detected SVs were merged by Survivor in such a way that variants occurring close to each other were not merged by the program. This was achieved by setting the maximum allowed distance between merged SVs to 1 bp. We also used only SVs longer than 50 bp for the following analyses.

### Formal definitions

In this work, the SV data is represented by a genotype matrix with $N$ rows corresponding to individual SVs and $M$ columns corresponding to individual probands. The elements of the matrix correspond to the genotypic state of the variant in a given individual. We distinguished three genotypic states: $0$ represents the reference homozygous genotype, $1$ represents the heterozygous genotype, and $2$ represents the alternative homozygous genotype.

Every SV can then be represented by a genotype vector $v_{i}$ of length $M$, containing a genotype state of $i$-th SV for every individual, where $i = 1, \ldots ,N$.

We defined the merging of any subset of SVs as a simple summation: $\mathop \sum \nolimits_{{i \in {\varvec{s}}}} v_{i}$, where ${\varvec{s}} \subseteq \left\{ {1, \ldots ,N} \right\}$.

We call SV pair with corresponding vectors $v_{i}$ and $v_{j}$ “merging-incompatible” if $k \in \left\{ {1, \ldots ,M} \right\}$ exists, where $v_{i} { }\left( k \right) \ne 0$ $\wedge$ $v_{j} { }\left( k \right) \ne 0$ and $i,j \in \left\{ {1, \ldots ,N} \right\}$.

To demonstrate the concepts used in this work, we also represented separately the genotypic state of the $i$-th SV in the members of any parent–child trio by a vector $u_{i}$ of length three, with the convention that the child genotype appears at the first position.

We call $u_{i}$ non-trivial if $l \in \left\{ {1,2,3} \right\}$ exists, where $u_{i} { }\left( l \right) \ne 0$.

### Algorithms for SV clustering

The differences between commonly used clustering strategies lie mainly in the definition of the dissimilarity measures used for clustering SVs of the same type. Two basic definitions are widely used. The first is defined as a function of overlap between genomic regions corresponding to SV pair [4, 7]. We used the following form in this work:

$$D_{1} = 1 - \frac{{\left| {g_{1} \cap g_{2} } \right|}}{{\max \left( {\left| {g_{1} } \right|,\left| {g_{2} } \right|} \right)}}$$

where $g_{1}$ and $g_{2}$ are genomic intervals corresponding to two SVs.

The second is the maximum of two distances between the starting and ending position of the SV pair [6]. We used the following form in this work:

$$D_{2} = \max \left( {\left| {{\text{start}}\left( {g_{1} } \right) - {\text{start}}\left( {g_{2} } \right)} \right|,\left| {{\text{end}}\left( {g_{1} } \right) - {\text{end}}(g_{2} } \right|} \right)$$

where ${\text{start}}\left( {g_{1} } \right)$ denotes the starting genomic position of $g_{1}$ and ${\text{end}}\left( {g_{1} } \right)$ denotes the ending genomic position of $g_{1}$, analogically for $g_{2}$.

With the definition of the dissimilarity measure, the clustering procedure is straightforward: we must select a threshold value ($D_{max}$) and then find components of the graph defined by pairs with a value of $D_{1\left( 2 \right)} \le D_{max}$. These components are formed by SVs that we assume correspond to a single SV detected with slightly different breakpoints within different samples. All SVs forming the component are then merged into one.

There is one ambiguity at this point that must be considered. It could happen that during the merging of two SVs, both are presented within a single sample in a non-reference genomic state. It is ambiguous what the resulting genotype should be for this sample after merging both SVs. We call a pair of SVs that cannot be unambiguously merged “merging-incompatible” in the article—see the “Formal definitions” section above. Surprisingly, this problem is not mentioned in the studies, where SV clustering is used to merge large numbers of samples. According to our knowledge, there are two possible explanations for this: 1) these two SVs are on different strands of DNA, or 2) both are on the same strand. It follows that the above-mentioned SVs represent different variants detected within a single sample and should not be merged.

A simple solution to this situation may be not to merge SVs in the cluster that show the presence of a merging-incompatible SV pair. We presented the results of this strategy within the article for the sake of completeness. We refer to this as “trivial strategy.”

We also used this more convenient strategy to solve the above-mentioned problem.

Let us assume we obtained components for some value of $D_{max}$.

1. Find all components where at least one merging-incompatible SV pair exists.
2. For every component defined in step 1, do the following:
  - Find maximal $D < D_{max}$, where all SVs from the component are distributed within sub-components that do not contain any merging-incompatible SV pairs.
  - Replace the component with set of sub-components identified in step 2a.

We refer to this strategy as “corrected strategy.”

### New algorithm for SV clustering

#### Combinatorial search of all SV pairs and triplets that can be merged into a Mendelian-consistent single SV

Mendelian-consistent SVs can only be created by merging two or three SVs represented by a non-trivial vector $u$. The addition of any other non-trivial vector $u$ to the triplet must result in a merging-incompatible SV pair (see section “Formal definitions”). This fact ensures the computational feasibility of the combinatorial search.

From the combinatorial viewpoint, 26 distinct vector pairs $u$ existed, resulting in Mendelian-consistent SVs after merging. In contrast, there were only seven distinct vector triplets $u$ with the same properties.

The algorithm searches for all the pairs and triplets (groups) within every trio and tests the following two conditions for every identified group of SVs:

1. The group of SVs cannot be merging-incompatible if we consider all samples (not only trio members).
2. $D_{1\left( 2 \right)} \le D_{max}$ must hold for every pair of SVs within the group.

Only groups meeting both criteria are considered for the next step of the algorithm.

#### Reduction to disjoint set of SV groups

It is theoretically possible that the algorithm could identify two non-disjoint groups of SVs within a single trio, both satisfying the criteria defined in the previous section. It follows that these two groups are merging-incompatible, so we must decide which group to retain for further analysis. We implemented the following heuristic strategy to deal with intersecting SV groups.

At the first step we represented the identified superset of SV groups as an un-oriented graph, where every identified SV group corresponded to a unique vertex. The edge between two vertices exists if the corresponding SV groups share at least one SV (i.e., having a non-zero intersection). We then identified all connected components within the graph and applied the following procedure on every such component, $c_{i}$:

1. Select SVs group from $c_{i}$ which is detected in highest number of trios or randomly if all groups are equal.
2. Test if the given group is merging-compatible with already merged groups from $c_{i}$ and if the merging results in a Mendelian consistent SV in all trios.
3. Delete the group from the queue. If the test in step 2 succeeds, merge the group with already merged groups from $c_{i}$ and add the group into the newly formed reduced component, $c_{ir}$, where $c_{ir} \subseteq c_{i}$.
4. Return to step 1.

#### Constrained clustering

We constructed another type of graph where vertices correspond to individual SVs (and not the SVs groups as in the previous case). The edge exists between two SVs if both belong to any reduced component ($c_{ir}$).

Another required ingredient of constrained clustering is a cannot-link matrix corresponding to the logical triangular matrix that contains which SV pairs are merging-incompatible.

Constrained clustering can be described by the following procedure:

1. Select all pairs of SVs with a minimum value of $D_{1\left( 2 \right)}$ that are not part of any single component.
2. For every pair of SVs identified in the previous step do the following:
  - Merge the two components to which the examined SV pair belongs and test if the newly formed component contains any merging-incompatible SV pairs.
  - If the test in 2a. fails, accept the new edge connecting the investigated SV pair.
3. After all pairs with an actual minimum value of $D_{1\left( 2 \right)}$ are examined, delete them from the queue and return to step 1.

We refer to this strategy as a “constrained clustering strategy.”

### Measures used for SV dataset quality evaluation

#### Mendelian inheritance error

The first measure used for clustering quality evaluation was the fraction of SVs corresponding to Mendelian errors $\left( {f_{MEI} } \right)$. Mendelian inheritance error (MIE) represents the combination of a child’s and its parents’ three genotypes that are inconsistent with Mendelian inheritance. We computed $f_{MEI}$ for every trio and then averaged it over families ${\mathbf{A}}$ and ${\mathbf{B}}$. There is general agreement that most Mendelian-erroneous genotype configurations are caused by sequencing/algorithm detection errors. Only a tiny fraction of Mendelian errors (where parents’ genotypes correspond to the reference homozygotes and the child genotype corresponds to an alternative heterozygote) can be caused by de-novo mutation.

#### The number of Mendelian-consistent SVs resulting from merging SV clusters exhibiting Mendelian errors

We defined the new measure of SV dataset quality on the assumption that the Mendelian errors exhibit a specific pattern. We assumed that the observed Mendelian inconsistency can emerge as a product of erroneous determination of SV position in one or more of a trio’s members. The single SV will then be represented as two or three different SVs detected in slightly different positions. As a result, one or more alleles may be missing in the genotype configuration of the family trio because it is erroneously detected as a different SV with a different position. We will demonstrate this concept using formalism established above.

Let us assume we have a parent–child trio with an SV corresponding to Mendelian-consistent genotype configuration $\left( {2,1,1} \right)$. If this SV is detected in one of the parents in a slightly different position, we will observe two neighboring SVs with the following genotype configurations: $\left( {2,1,0} \right)$ and $\left( {0,0,1} \right)$. We can see that the first configuration represents Mendelian error and the second is Mendelian consistent. Both configurations can be unambiguously merged into a single Mendelian-consistent SV.

According to this concept, we propose the following quantity to measure SV dataset quality: the number of Mendelian-consistent SVs resulting from merging SV clusters exhibiting at least one MEI.

#### Kinship prediction

We used the R package Demerelate [12] to predict kinship categories for two families within our dataset. We used two different estimators: the Loiselle coefficient [13] and the proportion of shared alleles $\left( {S_{xy} } \right)$ [14]. The Loiselle coefficient represents a more complex measure that considers population SV frequencies estimated from non-related individuals within our dataset and corrects for a small sample size. The Loiselle coefficient, therefore, reflects the quality of the whole data set. In contrast, $S_{xy}$ represents a simple measure based only on allele sharing between paired individuals. We presented the results obtained with $S_{xy}$ only in Additional file 1 because they are similar to the results obtained with the Loiselle estimator.

We used the error related to the ability with which both estimators can separate individual kinship categories as a measure of dataset quality. Let us assume we have $n$ kinship categories $i = 1, \ldots ,n$ corresponding to relationship coefficients $r_{i}$, where $r_{i} < r_{i + 1}$ for $i = 1, \ldots ,n - 1$. We computed the following quantity for every kinship category:

$$L_{i} = \mathop \sum \limits_{{\begin{array}{*{20}c} {j < k} \\ {j,k \in i} \\ \end{array} }} \delta \left( {j,k} \right)$$

where $\delta \left( {j,k} \right) = 1$ if $C_{i} \left( {j,k} \right)\left\langle {\min \left( {C_{i + 1} } \right) \wedge C_{i} \left( {j,k} \right)} \right\rangle \max \left( {C_{i - 1} } \right)$, otherwise $\delta \left( {j,k} \right) = 0$ and where $C_{i} \left( {j,k} \right)$ denotes the estimated value of relatedness for individuals $j$ and $k$, and ${\text{max}}\left( {C_{i} } \right)$ denotes maximum estimated value within kinship category $i$, analogically for $\min \left( {C_{i} } \right)$. On the basis of quantity $L_{i}$, we can define the error rate for every kinship category as:

$$s_{i} = 1 - \frac{{L_{i} }}{{\left( {\begin{array}{*{20}c} {\left| i \right|} \\ 2 \\ \end{array} } \right)}}$$

where $\left| i \right|$ denotes the number of pairs of individuals within the kinship category $i$. For the purpose of comparing SV clustering methods, we used the average value of $s_{i}$ across all kinship categories presented within the pedigree, $\left\langle s \right\rangle = \frac{1}{n}\mathop \sum \limits_{i} s_{i}$.

### Deviation from Hardy–Weinberg equilibrium

Deviation from the Hardy–Weinberg principle is a widely used measure for quality evaluation of datasets containing population genetic variants. We followed the same methodology as applied in the work of Karczewski et al. [4]. We computed the chi-square *p*-value using the Hardy–Weinberg R package and applied the Bonferroni correction. Those SVs with *p* < 0.05 after the Bonferroni correction were considered to violate the Hardy–Weinberg equilibrium. To compare the SV clustering methods, we used the fraction of SVs that are in Hardy–Weinberg equilibrium.

### Randomization of SV distribution within clusters

We generated an ensemble of only ten randomized samples for the corrected clustering strategy for each dissimilarity measure ($D_{1}$ and $D_{2}$). We generated only 20 randomizations in total due to the high computational complexity. The main purpose of the random model within this work was to exclude the null hypothesis that the behavior of the quantities used for dataset quality evaluation is determined by randomness, and, thus, their importance in clustering method comparisons is limited. Our goal during the randomizations was to preserve the size distribution of clusters before the correction for merging-incompatible SVs (as defined for corrected strategy). We performed randomizations by reshuffling the rows of the genotype matrix associated with each SV type. This procedure is equivalent to randomly partitioning SVs of the same type into clusters having the identical size distribution as exhibited by the real data. The randomization was performed before the correction because we needed to ensure that merging-incompatible SV pairs would not be presented within the final clusters.

## Supplementary Information

**Additional file 1**. Average degree of separation between different kinship categories achieved by *S*_*xy*_ kinship estimator.

## Data Availability

The initial dataset of SVs used during the current study is available from the corresponding author on reasonable request. The computer code for the constrained clustering algorithm written in R programming language is freely available at: https://github.com/geryk/Constrained-clustering-of-structural-variants [11].
