# Supplementary material for: Improving structural variant clustering to reduce the negative effect of the breakpoint uncertainty problem
Source: BMC Bioinformatics. 2021 Sep 27;22:464. doi: 10.1186/s12859-021-04374-3 (PMC8474851; doi:10.1186/s12859-021-04374-3)
Supplement: Supplementary file 1 — Additional file 1. Average degree of separation between different kinship categories achieved by Sxy kinship estimator. [file 12859_2021_4374_MOESM1_ESM.docx]

Average degree of separation between different kinship categories achieved by $S_{xy}$ kinship estimator.


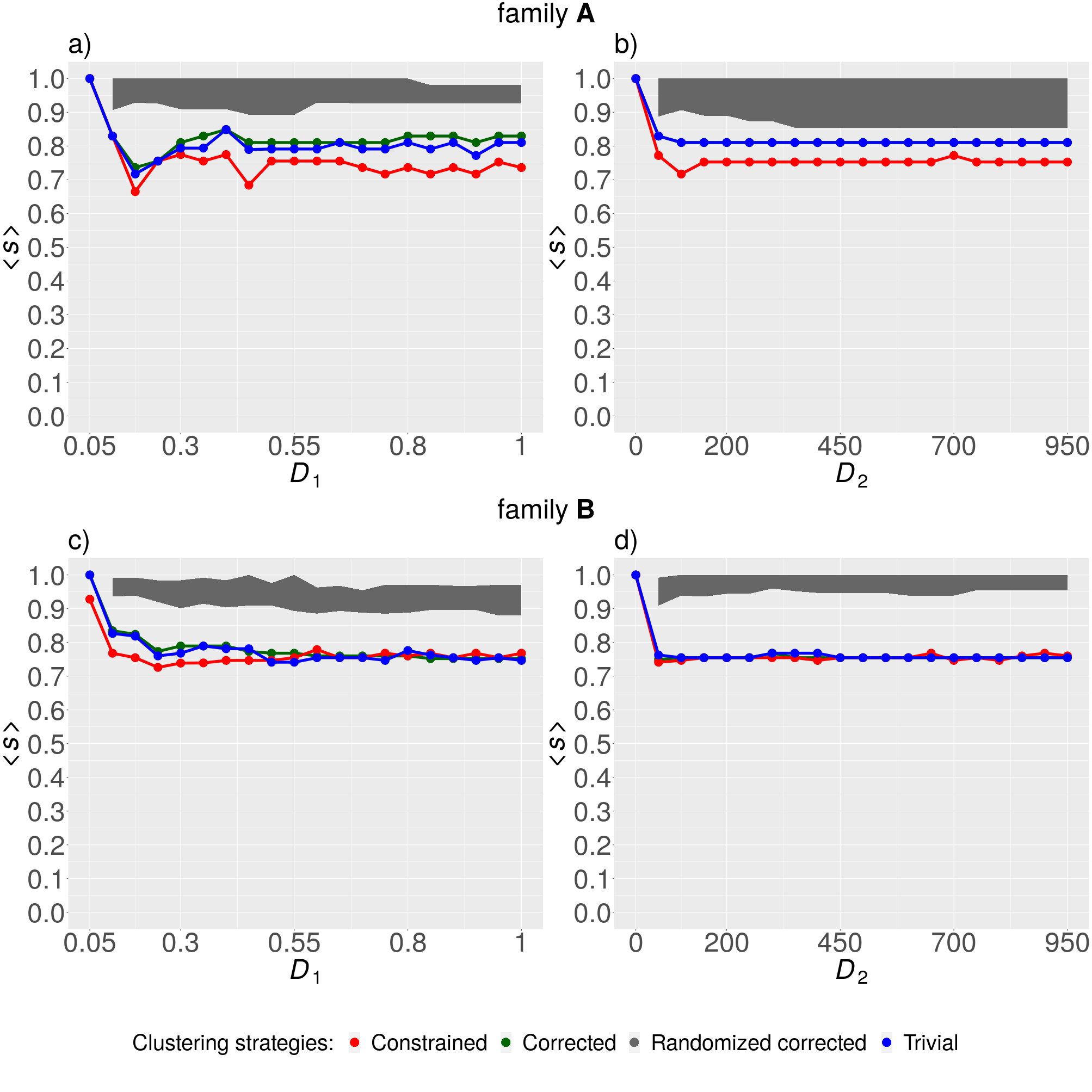


Fig. 6. Average degree of separation between kinship categories achieved by $S_{xy}$ estimator $\left\langle s \right\rangle$.

The dependence of $\left\langle s \right\rangle$ on the value of $D_{1}$ in family A (a) and family B (c) and the dependence of the same quantity on the value of $D_{2}$ in family A (b) and family B (d). Random ensemble is represented by the gray area defined from above by maximum values of $\left\langle s \right\rangle$ and from below by minimum values of $\left\langle s \right\rangle$.
